# Recipient screening in IVF: First data from women undergoing anonymous oocyte donation in Dublin

**DOI:** 10.1186/1742-4755-8-8

**Published:** 2011-04-20

**Authors:** Anthony PH Walsh, Ahmed B Omar, Kevin D Marron, David J Walsh, Umme Salma, E Scott Sills

**Affiliations:** 1 Division of Reproductive Endocrinology, The Sims Institute (Dublin)/Sims IVF, Department of Obstetrics & Gynaecology, School of Medicine, Royal College of Surgeons in Ireland; Dublin Ireland; 2 The Sims Institute, Rosemount Hall, Dundrum, Dublin 14 Ireland

## Abstract

**Background:**

Guidelines for safe gamete donation have emphasised donor screening, although none exist specifically for testing oocyte recipients. Pre-treatment assessment of anonymous donor oocyte IVF treatment in Ireland must comply with the European Union Tissues and Cells Directive (Directive 2004/23/EC). To determine the effectiveness of this Directive when applied to anonymous oocyte recipients in IVF, we reviewed data derived from selected screening tests performed in this clinical setting.

**Methods:**

Data from tests conducted at baseline for all women enrolling as recipients (*n *= 225) in the anonymous oocyte donor IVF programme at an urban IVF referral centre during a 24-month period were analysed. Patient age at programme entry and clinical pregnancy rate were also tabulated. All recipients had at least one prior negative test for HIV, Hepatitis B/C, chlamydia, gonorrhoea and syphilis performed by her GP or other primary care provider before reproductive endocrinology consultation.

**Results:**

Mean (±SD) age for donor egg IVF recipients was 40.7 ± 4.2 yrs. No baseline positive chlamydia, gonorrhoea or syphilis screening results were identified among recipients for anonymous oocyte donation IVF during the assessment interval. Mean pregnancy rate (per embryo transfer) in this group was 50.5%.

**Conclusion:**

When tests for HIV, Hepatitis B/C, chlamydia, gonorrhoea and syphilis already have been confirmed to be negative before starting the anonymous donor oocyte IVF sequence, additional (repeat) testing on the recipient contributes no new clinical information that would influence treatment in this setting. Patient safety does not appear to be enhanced by application of Directive 2004/23/EC to recipients of anonymous donor oocyte IVF treatment. Given the absence of evidence to quantify risk, this practice is difficult to justify when applied to this low-risk population.

## Introduction

Since 2006, the European Union Tissues and Cells Directive (Directive 2004/23/EC) has exerted an important regulatory impact on the provision of clinical fertility practice in the E.U [1]. This legislation was designed to ensure the safety of donated tissue including bone marrow, corneas, stem cells and heart valves provided through organ/tissue donation. However, IVF clinics in Europe found that they too were under the remit of this directive, since, from a technical and legislative standpoint, couples were regarded as "donating" their own eggs and sperm to each other during fertility procedures. E.U. member states have applied this testing requirement to their constituent IVF clinics in different ways. This process continues to evolve in the Republic of Ireland, and accumulated evidence is beginning to show that there is room for improvement in how IVF patients are screened before treatment [2].

Studies of screening test results have found the current testing approach has a very poor yield when applied to non-donor IVF cycles in Ireland [3]. Indeed, anonymous oocyte donors matched to recipients in Ireland were confirmed to be low-risk as well [1]. But until now, there has not been any detailed analysis of basic screening data from recipients of anonymous donor oocyte IVF cycles in Ireland. The current investigation describes these Irish recipients of anonymous donor oocyte IVF treatment with specific attention to their HIV, Hepatitis B/C, chlamydia, gonorrhoea, and syphilis test results.

## Methods

### Study population and specimen collection

Written informed consent was obtained from all patients (*n *= 225) who enrolled in the anonymous donor oocyte IVF sequence at The Sims Institute (Dublin)/Sims IVF during a 24-month period ending January 2011. Because the study site is the only provider of anonymous donor oocyte IVF service in the Republic of Ireland, the geographical catchment area includes the entire state (patients from other jurisdictions also obtained treatment here, but data from foreign individuals were not stratified for separate analysis). All donor-egg IVF cycles reviewed in this study incorporated sperm provided by a known partner who was also separately evaluated, rather than from an anonymous sperm donor [4]. All participants underwent formal psychological counselling in advance of anonymous donor oocyte IVF and each patient received standard clinical examination. Tests for Chlamydia and syphilis were done either by serological (blood test) method, cervical swab, or urine test in compliance with Directive 2004/23/EC. In contrast, this directive does not require separate testing for *N. gonorrhoea *although this test was performed based on current ASRM testing guidelines for oocyte recipients in the United States [5]. All specimens were processed at a registered INAB licensed laboratory accredited to ISO medical testing standards. Blood samples were collected in appropriate vacutainer tubes in a non-fasting, random state via peripheral venipuncture (sample volume 5-10 ml). Urine samples were collected at least 2 h after previous urination (sample volume = 10-20 ml). Cervical swabs were collected at pre-enrolment assessment during speculum exam at this centre. All specimens were sent to our diagnostic laboratory, transported neat for examination within 48 h.

### Overview of specific testing platforms

#### Chlamydia

Testing was via PCR test performed either on cervical swab or random urine sample (these testing modalities have been determined to be of equivalent accuracy and sensitivity) [6]. The test amplifies a fragment of species specific DNA from the plasmid of the *Chlamydia *organism which is present in the female reproductive tract, and has sufficient sensitivity to detect a single chlamydia cell in the sample submitted for analysis. The PCR test for *C. trachomatis *had no cross-reactivity with antigens from *C. pneumoniae *or *C. psittaci *(psittacosis), which require different detection methods.

#### Gonorrhoea

Urine samples were used to detect bacterial *N. gonorrhoeae *DNA via spin-column chromatography and PCR amplification. The amplified PCR products were then further studied by agarose gel electrophoresis. This test incorporates reagents and enzymes for specific amplification of a 260 bp region of *N. gonorrhoeae's *PorA gene. A second heterologous amplification system is also used to identify possible PCR inhibition and/or inadequate isolation.

#### Syphilis

Peripheral venipuncture was used to collect samples which were used with an enzyme immunoassay (EIA) method to detect IgM or IgG class antibodies (Abs) directed against *T. pallidum*. This protocol involves application of highly specific recombinant syphilis antigens for Abs that arise early during the primary stage of syphilis, and have lifelong positivity in most cases.

#### Hepatitis B & C, HIV

Microparticle enzyme immunoassay (MEIA) techniques were used for qualitative detection of Hepatitis B surface antigen (HBsAg) in human serum as well as detection of antibodies to Hepatitis B core protein (anti-HBc). All samples were run on an AxSYM Plus diagnostic platform (Abbott Diagnostic Division Ltd., Abbott Park, Illinois USA) utilizing microparticles coated with monoclonal antibodies for the detection of the above markers. Samples non-reactive by this testing modality are considered negative for Hepatitis B and need not be tested further. MEIA was also used for qualitative detection of antibody to hepatitis C virus (anti-HCV) in human serum. Similarly, simultaneous qualitative detection of antibodies to human immunodeficiency virus type 1 and/or type 2 (HIV-1/HIV-2) as well as the HIV p24 antigen in human serum was also performed. This global HIV assay (AxSYM HIV Ag/Ab Combo) does not, however, discriminate among HIV-1 antibody, HIV-2 antibody, or HIV p24 antigen reactivity. For these tests, assay result was determined by comparing fluorescent product formation rate to a cut-off rate, derived from previous AxSYM Index Calibrations. The sample is classified as reactive if the rate of formation of fluorescent product in specimens is greater than or equal to this threshold.

### Analysis and follow-up protocol

In addition to results from the screening tests above, female patient age and clinical pregnancy rate (per embryo transfer) were recorded. Validation is required for any positive screening result, with reflex testing performed at the National Virus Reference Laboratory (UCD-Ireland).

## Results

In the 24-month period ending January 2010, the mean (±SD) age for 225 consecutive patients who enrolled in anonymous donor oocyte IVF programme was 40.7 ± 4.2 yrs. At least one referring (primary care) physician had provided negative test results for HIV, Hepatitis B/C, chlamydia, gonorrhoea, and syphilis prior to reproductive endocrinology consultation and IVF (average interval between GP screening and re-screen = 31 months; range 2 - 60 months).

To comply with Directive 2004/23/EC as interpreted by Statutory Instrument (SI) 158 of 2006, SI 598 of 2007, a 2007 IMB Draft, and other IMB communications, HIV, Hepatitis B/C, chlamydia, gonorrhoea, and syphilis tests were again carried out at our centre and designated "baseline studies". All test results were reviewed first by clinic personnel with secondary verification by staff at the commercial laboratory responsible for sample processing. All specimens were marked as satisfactory for analysis and no positives were identified for any specimen submitted. Among patients undergoing anonymous donor oocyte IVF, the mean clinical pregnancy rate (per embryo transfer) during the study interval was 50.5%. All patients completed IVF without complications and there were no adverse events reported among recipients or anonymous oocyte donors.

## Discussion

In recent years, mean age of infertility patients at initial consultation appears to have increased and has been accompanied by a sharp rise in the number of prior failed IVF cycles [7]. This refractoriness can be expected to result in higher interest in anonymous oocyte donation IVF, although how best to screen these oocyte recipients is not known with certainty. In Europe, regulations detailing mandatory testing for any IVF patients are lacking. The ESHRE Task Force on Ethics and Law (2002) carefully studied the matter of gamete and embryo donation, but did not recommend specific testing requirements for recipients. The issue of oocyte donation was addressed in more detail in the European Union Tissues and Cells Directive (Directive 2004/23/EC) [8], yet testing guidelines for recipients of donated oocytes were again omitted. While this directive was designed to assure medical consumers of the safest possible healthcare rendered at the best possible standards, how exactly this objective should be applied to oocyte recipients remains undefined. Europe's key opinion leaders in reproductive medicine have questioned the appropriateness of applying this directive to general IVF practice [9], and have highlighted the low yield (and increased patient expense) associated with an unnecessarily broad screening approach.

In Ireland, investigations focusing on screening data from non-donor IVF patients have likewise concluded that such individuals constitute a very low risk group, where the level of disease surveillance in this subgroup should be different compared to the general population. For Irish IVF patients and the physicians who offer this treatment here, the challenge to define proper testing is particularly acute given the absence of any legislation dealing with assisted reproductive techniques here [10]. The current report is the first to aggregate screening data from recipients who underwent anonymous donor oocyte IVF in Ireland; no positive screens were identified among 225 consecutive IVF cases completed during the study interval. While our results are derived from a particular sub-group of IVF patients (*i.e.*, anonymous donor oocyte recipients), these findings agree with general pre-fertility treatment screening data independently published from another leading Irish IVF centre [3] where all test subjects also had negative screens. Taken together, these factors make it increasingly difficult to justify the current screening approach for IVF patients in Ireland as mandated by Directive 2004/23/EC.

Making sure patients have no serious infection before commencing fertility treatment is important. But since patients in our study all had negative tests performed with their GP or other primary care provider before embarking on IVF treatment, our (repeat) screening provided no additional clinically relevant information. We can therefore endorse the general view that re-screening of IVF patients in Ireland is not an effective method to enhance patient safety [3]. Indeed, our study permits refinement and extension of their conclusions specifically to anonymous donor oocyte IVF recipients, where repeat HIV, Hepatitis B/C, syphilis, gonorrhoea, and chlamydia tests also yielded nothing but increased patient cost and potential anxiety. We believe that if such testing has already been carried out on the oocyte recipient in a previous (primary care) setting with negative results, then this is sufficient *a priori *evidence that "best practice standard" has been met. Continued use of Directive 2004/23/EC to justify repeat testing for HIV, Hepatitis B/C, syphilis, gonorrhoea, and chlamydia specifically among recipients of anonymous donor oocyte IVF treatment is unwarranted in the absence of medical evidence to quantify risks for this patient population.

## Competing interests

The authors declare that they have no competing interests.

## Authors' contributions

APHW was the lead investigator and directed data aggregation, ABO, DJW and US were consultants who supervised patient screening and participated in data collection, KDM directed laboratory operations and ESS developed the project and coordinated the manuscripts. All authors read and approved the final manuscript.
